# Outcomes of anatomic total shoulder arthroplasty: evaluation of implant-related, radiographic, and demographic factors influencing durability and revision rates

**DOI:** 10.1007/s00264-025-06454-y

**Published:** 2025-03-01

**Authors:** Felix Hochberger, Benedikt Weth, Tizian Heinz, Dirk Boehm, Maximillian Rudert, Kilian List

**Affiliations:** 1 Department of Orthopaedic Surgery, University of Wuerzburg, Koenig-Ludwig-Haus, Brettreichstr 11, 97074, Wuerzburg, Germany; 2 Ortho Mainfranken, Wuerzburg, Germany

**Keywords:** Follow-up assessment, Shoulder prosthesis, Head-stem index, Osteolysis

## Abstract

**Purpose:**

To evaluate the impact of implant-associated and radiographic factors on survival rates and revisions of total shoulder arthroplasty (TSA) in patients with primary osteoarthritis (OA).

**Methods:**

This retrospective study included 68 patients who underwent TSA for primary OA at a single institution between 2008 and 2015, with a minimum follow-up of 60 months. Patients with prior shoulder surgeries, perioperative infections, or revisions within 12 months postoperatively were excluded. Patients were divided into Group A (Survivors) and Group B (Revisions) based on implant survival. Radiographic parameters analyzed included critical shoulder angle (CSA), acromiohumeral distance (AHD), lateral offset (LO), humeral head-stem index (HSI), centre of rotation (COR), and glenoid erosion, categorized using Sirveaux, Lévigne, Franceschi, and Walch classifications. Demographic data were also assessed.

**Results:**

Of 68 patients, 57 were in Group A (mean age: 58.5 ± 10.1 years; follow-up: 115.8 months) and 11 in Group B (mean age: 61.4 ± 8.3 years; follow-up: 113.9 months). Implant survival was 84% after 115.8 ± 34.5 months. Baseline demographics were comparable (e.g., smoking: *p* = 0.75), as was osteolysis prevalence (Group A: 47%; Group B: 45%; *p* = 0.91). HSI was significantly higher in Group B (0.5 ± 0.1 vs. 0.4 ± 0.1; *p* = 0.03). No other radiographic differences were significant.

**Conclusion:**

Patients undergoing anatomic total shoulder arthroplasty can expect favourable mid- to long-term outcomes, with implant survival rates of 84% and relatively low complication rates. Although osteolysis is common, it rarely necessitates revision surgery. The role of the humeral head-stem index (HSI) warrants further investigation.

**Study design:**

Level IV; retrospective case study.

## Introduction

Anatomic total shoulder arthroplasty (TSA) is a well-established and dependable treatment for patients with advanced glenohumeral osteoarthritis (OA), adhering to the key principle of re-establishing the joint’s natural anatomy [1–3]. TSA has shown good to excellent outcomes in the mid- to long-term period, with notable pain relief and generally low complication rates [4–8]. Current literature reports survival rates exceeding 90% at 20 years postoperatively [9]. When revision surgery is required, loosening of the glenoid component, periprosthetic infection, and secondary rotator cuff insufficiency are the most common causes [6, 10–13]. The use of TSA is rising globally, driven by an aging population with increased physical demands in everyday life. Projections indicate that the demand for primary arthroplasty will surge by 333% between 2011 and 2030 [14]. This trend is particularly pronounced among the elderly, who have experienced the highest rate of increase in TSA incidence [15]. Since their initial introduction, ongoing advancements in surgical techniques and implant technology have led to progressively improved clinical and functional outcomes [6, 9]. Despite the trend towards shorter and even stemless humeral components, there is increasing interest in metal back glenoids and alternative options for conventional spherical head components, including the use of ellipsoidal head implants. While there are promising biomechanical results [16], clinical and radiological studies with a follow-up period of over two years are still awaited to better assess the advantages and disadvantages of modern implant technologies.

In recent years, reverse shoulder arthroplasty (RTSA) has also made a significant impact on improving clinical outcomes and extending the longevity of implants [17]. These advancements have greatly expanded its indications, creating substantial overlap with those traditionally associated with anatomic shoulder prostheses. Revision surgeries often lead to less favorable functional outcomes, increased soft tissue irritation, and diminished bone quality [18]. As a result, there is growing concern over the use of anatomic shoulder prostheses, particularly due to the risk of secondary rotator cuff insufficiency, which frequently necessitates revision surgery [19]. Some studies are now advocating for earlier consideration of reverse shoulder arthroplasty, even in cases where an anatomic prosthesis would traditionally be indicated [20, 21]. Given the increasing importance of implant longevity and revision rates, the aim of this study is to identify potential demographic, radiographic, and implant-associated factors that might help predict the mid- to long-term durability of TSA over a minimum follow-up period of five years.

## Materials and methods

### Patient selection

This retrospective study was conducted at a specialized high-volume centre for shoulder arthroplasty and included patients who underwent TSA between January 2008 and December 2015. The study focused on evaluating mid- to long-term outcomes, with a minimum follow-up period of 60 months. Inclusion criteria were adult patients (≥ 18 years) who received primary TSA for primary osteoarthritis of the shoulder (OA). Patients with a history of prior shoulder surgeries, perioperative infections and vascular or malignant diseases were excluded from the study. Moreover, patients with early revisions (within 12 months postoperatively) were excluded to focus on factors influencing mid- to long-term implant survivorship. Early revisions are typically related to perioperative complications rather than long-term implant performance. Patient-specific data, including demographics (age, sex, BMI, comorbidities) and surgical records, were retrospectively reviewed from our institution’s electronic medical records. A total of 68 patients met the inclusion criteria and were included in the final analysis (Fig. 4). This study was conducted in accordance with the principles outlined in the Declaration of Helsinki. Ethical approval was obtained from the Institutional Review Board of the University of Wuerzburg (Ref. 2020111101/14.12.2020), ensuring adherence to internationally accepted ethical standards for human research. Informed consent was obtained from all participants prior to data collection and analysis. Patient confidentiality was rigorously maintained throughout the study.

### Group allocation

At the end of the follow-up period, patients were categorized into two groups based on implant survival and revision rates: Group A (Survivors) and Group B (Revisions). An analysis of radiographic and implant-associated factors was conducted using preoperative and postoperative radiographs. Moreover, demographic data, including age, sex, BMI, smoking status, comorbidities (e.g., osteoporosis, diabetes, rheumatoid arthritis), and ASA scores, were compared between the groups to identify potential differences that could influence implant survivorship.

### Surgical management and postoperative rehabilitation protocol

All procedures were performed by experienced orthopaedic shoulder surgeons from the authors` institution using either the Arthrex Eclipse™ Total Shoulder Prosthesis System (Arthrex, Inc., Naples, Florida, USA) or the Tornier Simpliciti Shoulder System (Stryker, Inc., Kalamazoo, Michigan, USA). The arthroplasty procedure was conducted in accordance with the implant manufacturer’s guidelines. Cementation of the humeral component was determined based on patient-specific factors, intraoperative findings, and surgeon preference. Cementation of the PEG- or keeled glenoid component was performed in all patients, regardless of the implant selected. A deltopectoral approach was used in all cases, and the subscapularis (SSC) was managed using the peel-off technique in all cases and repaired with a transosseous double-row repair in every patient. The absence of the SSC was a contraindication to implant a TSA. Preoperative planning included a standardized radiographic assessment using radiographs, MRI, and CT scans. Intraoperative factors, such as cementation and any complications, were documented. Postoperatively, all patients adhered to a standardized rehabilitation protocol. The shoulder was immobilized in an abduction brace for six weeks, during which time passive and slight active-assisted mobilization was initiated. At seven weeks, patients progressed to active range of motion and strengthening exercises. Return to sports activities was permitted at four months postoperatively.

### Radiographic assessment

**Fig. 1:**
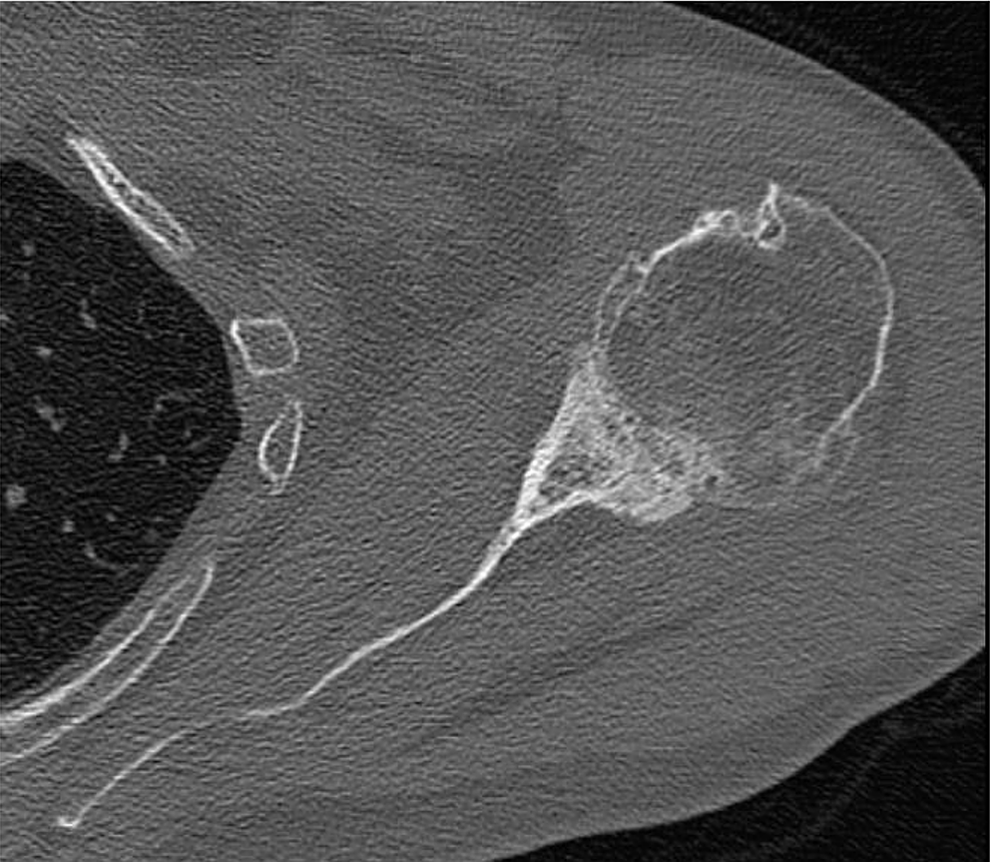
Preoperative axial CT-scan showing an A1 glenoid according to Walch et al. [25]

**Fig. 2:**
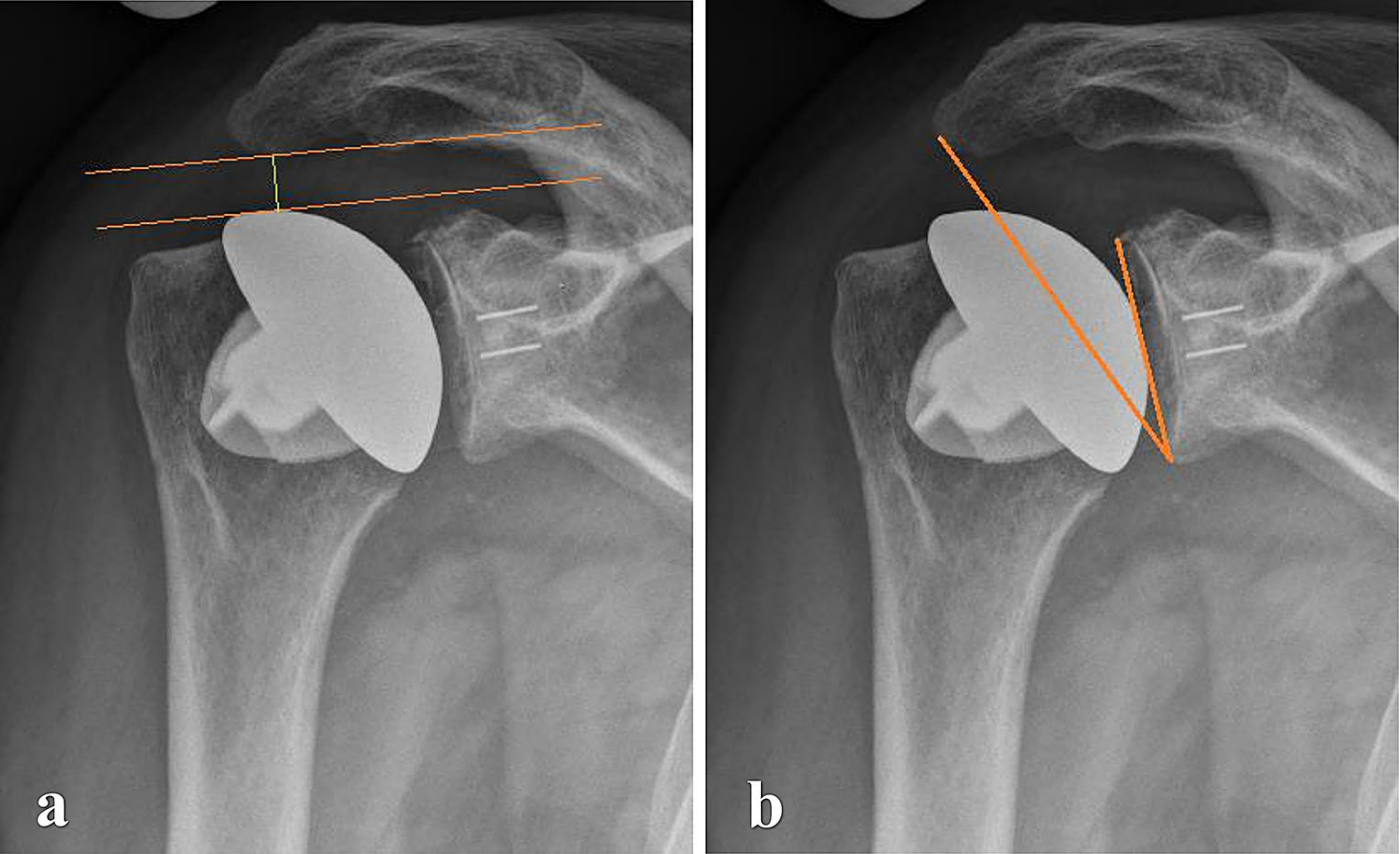
(**a**) Postoperative acromiohumeral distance (AHD, green line); (**b**) critical shoulder angle (CSA, angle demonstrated in orange)

**Fig. 3:**
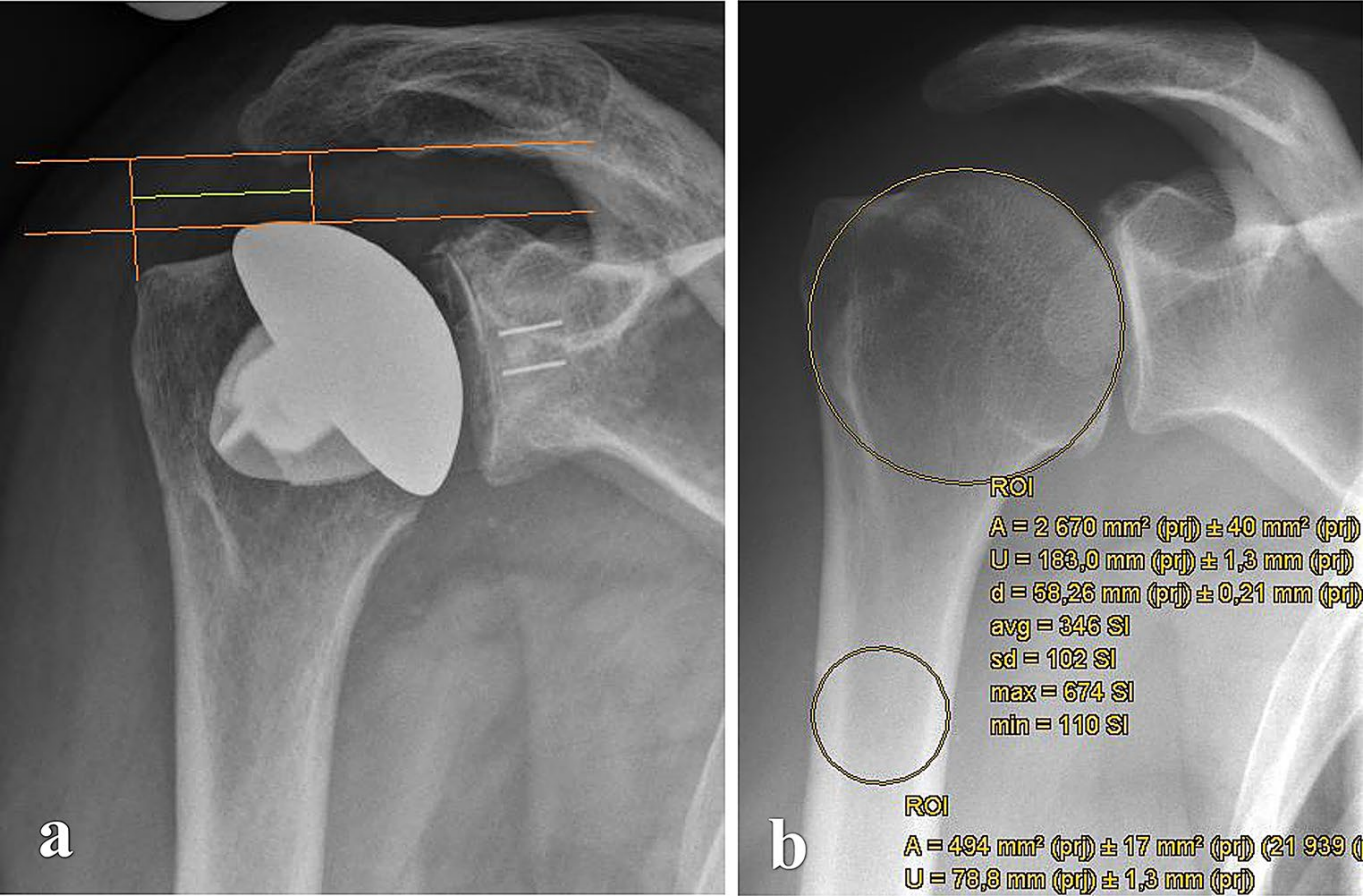
(**a**) Postoperative lateral offset (LO, green line); (**b**) preoperative humeral head-stem-index (HSI) (best-fit-circle technique)

Preoperative and postoperative radiographic assessments included true anteroposterior, axial, and Y-view images, which were also obtained during follow-up visits. In addition to these standard X-rays, preoperative imaging included computed tomography (CT) scans. Measurements on preoperative X-rays encompassed the critical shoulder angle (CSA) as described by Moor et al. [22], acromiohumeral distance (AHD), lateral offset (LO), head-stem-index (HSI), center of rotation (COR) and glenoid erosion categorized according to Sirveaux [23], Lévigne, and Franceschi [24]. Glenoid version was assessed using preoperative CT scans following the Walch classification [25] (Fig. 1). The AHD was measured as the perpendicular distance between the most lateral portion of the acromion’s undersurface and a line parallel to the superior border of the greater tuberosity, as outlined by Berthold et al. [26] (Fig. 2). CSA was determined by drawing a line from the superior to the inferior pole of the glenoid and another line from the inferior pole to the lateral edge of the acromion, based on the method described by Moor et al. [22] (Fig. 2). LO was measured on anteroposterior radiographs by determining the horizontal distance between the center of the humeral head and the perpendicular line drawn from the lateral edge of the acromion to the shaft of the humerus (Fig. 3). The humeral head diameter was determined by fitting a best-fit circle around the articular surface of the humeral head, with the circle’s diameter representing the humeral head size. The humeral shaft diameter was measured at the narrowest point of the medullary canal just below the metaphyseal region, and the Head-Stem Index (HSI) was calculated as the ratio of the humeral head diameter to the humeral stem diameter (Fig. 3). HSI is a novel concept in the context of shoulder arthroplasty, introduced in this study to explore proportional relationships between the humeral head and shaft dimensions. While the term ‘Head-Stem Index’ is derived from analogous measurements in hip arthroplasty, its application here pertains to the humeral shaft, as the index aims to provide insights into anatomical reconstruction during implant placement. The reliability of the HSI was evaluated by assessing inter- and intra-observer consistency, with two independent observers measuring the HSI on the same set of radiographs. evaluate COR was measured by fitting the best-matching circle to the articular surface to locate the center point within the humeral head. Subsequently, the distance was determined by measuring the perpendicular line from this center to the midpoint of the line connecting the superior and inferior glenoid tubercles [27]. Glenoid erosion was assessed on anteroposterior radiographs and classified according to the criteria established by Sirveaux, Lévigne, and Franceschi, which involves evaluating the extent of bone loss and the degree of medialization of the glenoid in relation to the scapular body [23]. To establish subgroups within Group B, the presence of osteolysis in the humerus and glenoid was used as the criterion. However, after forming the subgroups, the sample size (8 vs. 3) were too small, making a subgroup analysis infeasible. Radiographic assessment was conducted by two independent observers - one specialist with experience (FH) and one non-specialist (MW) - with results determined by consensus.

### Statistical analysis

Data analysis was conducted using SPSS software (version 27, SPSS Inc., Chicago, IL, USA). Ordinal variables were presented as means with standard deviations, while categorical variables were described using absolute or relative frequencies. The Kolmogorov–Smirnov test was employed to assess the normality of data distribution. Group differences were analyzed using either the independent *t*-test or the Mann–Whitney U test. Categorical variable frequencies were compared using the chi-square test. Within-group differences over time (preoperative to postoperative) were evaluated using the dependent *t*-test or Wilcoxon test. Additionally, logistic and linear regression analyses were performed to investigate the impact of several independent demographic and radiographic factors on the outcome variables, thereby adjusting for confounding variables. A priori sample size calculation was performed using G-power (version 3.1; Düsseldorf, Germany), assuming a conservative effect size and a statistical power of 0.8, which translated to a total sample size of 45 patients. A significance threshold was set at *p* < 0.05.

## Results

### Patient demographics

**Fig. 4:**
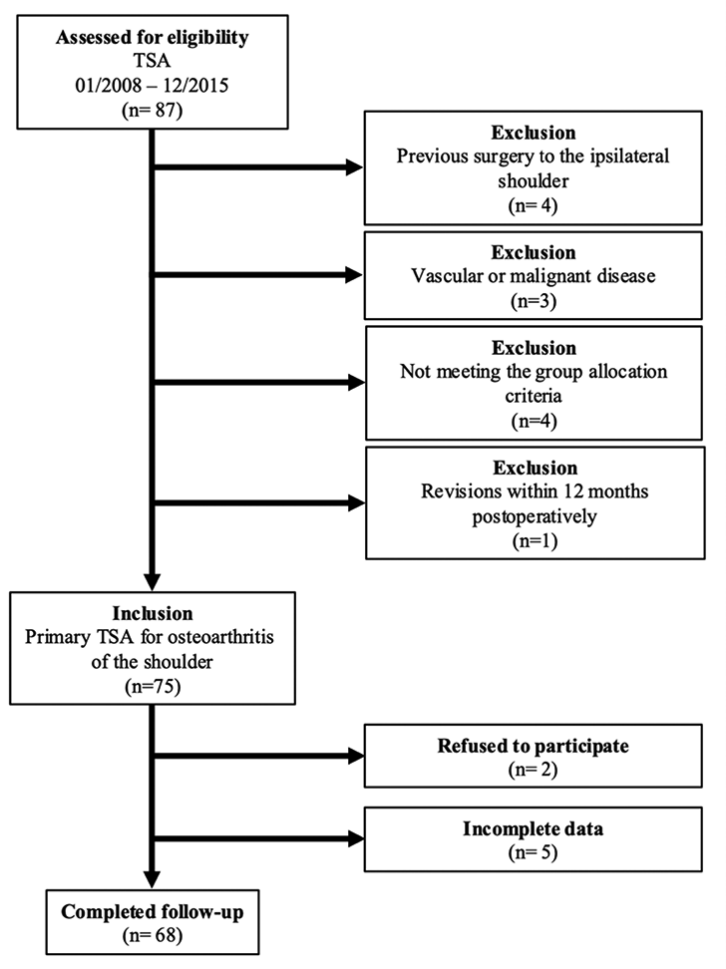
Flowchart displaying patients meeting study criteria

An analysis of the institutional database identified a total of 87 patients who underwent TSA between January 2008 and December 2015. Four patients were excluded due to previous surgery on the ipsilateral shoulder, three patients due to the presence of vascular or malignant disease, four patients were excluded for not meeting the group allocation criteria and one due to early revision surgery within 12 months postoperatively. Incomplete data were available for five patients, and two patients declined to participate in the study, leaving 68 patients available for evaluation (Fig. 4). Accordingly, the follow-up rate was 91%. Of these patients, 57 were assigned to Group A, as they did not undergo revision surgery by the end of the follow-up period, and 11 were assigned to Group B due to surgical revision. The groups did not significantly differ in terms of age, sex, BMI (body mass index), and ASA (American Society of Anesthesiologists) score (*p* > 0.05). Relevant comorbidities such as osteoporosis, diabetes mellitus, and rheumatoid arthritis were evenly distributed between Group A and Group B (*p* > 0.05). Additionally, the proportion of smokers was comparable in both groups (A: 23%, B: 27%; *p* = 0.75). In Group A, cementation of the humeral stem was performed in 14 patients (25%), while in Group B, it was noted in three patients (27%) (*p* = 0.85).

At the end of the follow-up period, osteolysis was present in 47% of cases in Group A and 45% in Group B, with no significant difference observed between the two groups (*p* = 0.91). All patients in Group B received surgical revision to a reverse total shoulder arthroplasty. The main cause for revision surgery in our cohort was aseptic glenoid loosening (*n* = 6), followed by secondary rotator cuff failure (*n* = 3). The remaining two patients in Group B experienced both aseptic glenoid loosening and secondary rotator cuff failure. Baseline demographics and patient characteristics of the two groups are summarized in Table 1.

**Table 1:** Table displaying demographics and patient characteristics of the groups

	Group A (survivor)	Group B (revision)	*p*-value
Total number, n	57	11	< 0.01
Age, y.	58.5 ± 10.1	61.4 ± 8.3	0.38
Sex, m/f	28 / 29	4 / 7	0.44
BMI, kg/m^2^	30.4 ± 6.1	30.4 ± 4.8	0.98
Follow-up, m	115.8 ± 34.5	113.9 ± 27.7	0.87
ASA Score			0.11
I	10	0	
II	2	9	
III	19	2	
IV	0	0	
Secondary Diagnosis			
Osteoporosis	5 (9%)	1 (9%)	0.97
Diabetes	7 (12%)	3 (27%)	0.20
Rheumatic disease	5 (9%)	0 (0%)	0.31
Smoker	13 (23%)	3 (27%)	0.75
Cementation	14 (25%)	3 (27%)	0.85
Osteolysis	27 (47%)	5 (45%)	0.91

### Radiographic outcomes

A univariate analysis demonstrated that a higher humeral head-stem index (HSI) both preoperatively and at the end of the follow-up period was a significant risk factor for revision and decreased implant durability. HSI was significantly higher in Group B (mean: 0.5 ± 0.1) compared to Group A (mean: 0.4 ± 0.1; *p* = 0.03). This difference, while statistically significant, reflects a small absolute variation of 0.04 between the groups. The potential clinical relevance of this finding is discussed further in the discussion section. The inter- and intra-observer reliability assessments for the HSI demonstrated no significant differences between observers, indicating consistent and reproducible measurements. None of the additional radiographic factors evaluated demonstrated a significant effect. Patients in Group B with an elevated HSI did not exhibit a correspondingly higher center of rotation (COR). The results of the radiographic assessment are presented in detail in in Table 2.

**Table 2:** Radiographic assessment. Normally distributed continuous variables are shown as mean ± standard deviation. Non-normally distributed continuous variables are shown as median (interquartile range)

	Group A (survivor)	Group B (revision)	*p*-value
CSA_pre	31.9 ± 5.2	31.1 ± 3.8	0.65
CSA_post	28.8 ± 5.2	28.8 ± 5.3	0.99
CSA_FU	32.0 ± 6.3	35.0 ± 6.7	0.17
LO_pre	10.8 ± 5.8	10.2 ± 4.9	0.75
LO_post	14.1 ± 7.2	14.6 ± 5.4	0.80
LO_FU	11.9 ± 6.3	9.7 ± 4.8	0.30
AHD_pre	10.0 ± 3.8	11.1 ± 3.3	0.35
AHD_post	14.0 ± 4.8	16.4 ± 4.8	0.14
AHD_FU	9.3 ± 4.3	9.7 ± 6.0	0.80
HSI _pre	0.4 ± 0.1	0.5 ± 0.1	0.03*
HSI _post	0.4 ± 0.1	0.5 ± 0.1	0.32
HSI_FU COR_pre COR_post	0.4 ± 0.1 27.6 ± 1.6 29.7 ± 0.3	0.5 ± 0.0 27.4 ± 2.3 27.7 ± 0.6	0.02* 0.34 0.22
Levigne_pre	2.0 ± 0.5	1.8 ± 0.4	0.32
Levigne_post	1.4 ± 0.5	1.5 ± 0.5	0.60
Levigne_FU	1.5 ± 0.5	1.3 ± 0.5	0.12
Sirveaux_pre	0.9 ± 0.6	0.6 ± 0.5	0.12
Sirveaux_post	0.5 ± 0.8	0.3 ± 0.5	0.30
Sirveaux_FU	0.9 ± 0.7	0.8 ± 1.2	0.82
Walch_pre	1.5 ± 0.7	1.3 ± 0.7	0.44

Significant differences are marked with *. *Abbreviations* CSA: critical shoulder angle; LO: lateral offset; AHD: acromiohumeral distance; COR: center of rotation; HSI: humeral head-stem-index; Levigne: glenoid erosion according to Lévigne and Franceschi; Sirveaux: glenoid erosion according to Sirveaux; Walch: glenoid morphology according to Walch; pre: preoperative; post: postoperative; FU: at the end of the follow-up period. The significance level was determined with *p* < 0.05

## Discussion

The key finding of this study suggests that a higher preoperative humeral head-stem index (HSI), as well as at the end of the follow-up period, may be linked to an increased risk of earlier revision of TSA during mid- to long-term follow-up. However, the absolute difference in HSI between the groups was only 0.04, with overlapping standard deviations (0.05 in both groups). While this difference was statistically significant, its clinical relevance remains uncertain. To our knowledge, no studies have yet examined the impact of the HSI on outcomes following TSA. HSI was measured on an anteroposterior radiograph and calculated as the ratio of the humeral head width and length to the humeral shaft width. A higher HSI may reflect the humeral head’s shift from a circular to a more ellipsoid, oval shape. A key factor in replicating the native humeral head anatomy is the discrepancy between prosthetic humeral head design and the actual structure of the proximal humerus. While most prosthetic humeral heads are designed with a circular shape, anatomical studies reveal that the humeral head is naturally ellipsoid, with the anterior-posterior dimension measuring about 2 mm less than the superior-inferior dimension [28–31]. It has been proposed that using a nonanatomic, circular prosthetic humeral head may apply excessive tension on the shoulder’s soft tissue envelope, potentially limiting postoperative ROM. Recent biomechanical research supports this idea, indicating that elliptical humeral heads can achieve better ROM outcomes than conventional circular designs in TSA [32, 33]. Therefore, it is plausible that there is an association between HSI and the restoration of the anatomical centre of rotation (COR). This assumption was not confirmed by the results presented here. The postoperative COR was classified as anatomical when the deviation from the preoperative state was less than 2.5 mm. Interestingly, Group B showed an almost unchanged COR (preoperative: 27.39 ± 2.33; postoperative: 27.74 ± 0.64), whereas Group A exhibited a slight increase in COR from 27.6 ± 1.63 preoperatively to 29.71 ± 0.25 postoperatively (Δ2.11 ± 1.65). According to our measurement criteria, an anatomical reconstruction of the COR was achieved in both groups. A recent study supports our measurement criteria, defining a deviation of more than 2.7 mm in COR from preoperative to postoperative in TSA patients as non-anatomical. The study also found that a deviation greater than 2.7 mm in COR is associated with reduced postoperative ROM following TSA [34]. Thus, achieving optimal postoperative ROM in TSA appears to depend on accurately restoring the humeral head’s native COR [35]. In our cohort, an elevated HSI did not result in a non-anatomical restoration of the COR. Additionally, our study observed a slightly higher proportion of female patients in the revision group (Group B) at 64%, compared to 51% in the survival group (Group A). However, this difference was not statistically significant (*p* = 0.44) and represents only a trend, suggesting a potential, but inconclusive, association between female sex and HSI. Clinically, we frequently note greater discrepancies between the humeral stem and head in female patients during surgery, though this observation remains anecdotal and lacks supporting data in current literature. Further studies are needed to investigate this hypothesis more thoroughly. Another significant finding was that a higher HSI, not only preoperatively but also at the end of the follow-up period, was associated with reduced implant longevity. Interestingly, no significant difference in HSI was observed between the groups immediately postoperatively. A potential explanation for this could be the use of a standardized surgical technique and implant selection criteria during the procedure, which likely minimized variations in humeral component placement at the time of surgery. Over time, however, factors such as progressive bone remodeling, micromotion at the bone-implant interface, or differential wear patterns might have contributed to the divergence in HSI observed at the end of the follow-up period. A greater mismatch between the glenoid surface and humeral head could over time also potentially intensify the “rocking horse” phenomenon [44], leading to increased asymmetric wear on the contact surface and compromising implant stability.

This study demonstrated an overall implant survival rate of 84% at a mean follow-up of nearly 10 years, confirming the durability of modern TSA implants. Notably, while the causes of revision in Group B, such as aseptic glenoid loosening (54.5%) and secondary rotator cuff failure (27.3%), were recorded, they were not analyzed further as this was not the primary focus of the present study. These findings, however, align with existing literature on TSA failure mechanisms [6].

Demographic comparisons between the two groups revealed no significant differences in age, sex, BMI, or comorbidities, suggesting that these patient-related factors did not significantly influence outcomes in our cohort. This observation is notable, as a review of the literature suggests potential differences in shoulder arthroplasty outcomes between men and women. Women are often reported to undergo shoulder arthroplasty at an older age, frequently presenting with greater preoperative disability and differing expectations compared to men [36]. Postoperatively, it is hypothesized that women may show lower functional scores and reduced range of motion, potentially due to differences in daily activities [36, 37]. Furthermore, women are thought to be more susceptible to periprosthetic fractures and aseptic loosening, while men are considered at greater risk for infections and revision surgeries [36]. In recent years, a marked increase in the proportion of female patients undergoing shoulder arthroplasty, particularly reverse shoulder arthroplasty, has been observed compared to male patients [38]. Consequently, it could be assumed that women might represent a larger proportion of those requiring revision surgery. However, current evidence does not establish female gender as a definitive risk factor for early revision in TSA [37].

None of the other radiographic or implant-related parameters demonstrated a significant effect on the longevity and revision rates following TSA, highlighting the complexity of predicting TSA outcomes and the multifactorial nature of implant survivorship. No increased CSA was observed in Group B compared to Group A, suggesting that CSA was not a risk factor for revision. These results contrast with the findings of Tabeayo et al. [39]. The authors showed in their matched control-trial of primary TSA that patients undergoing revision for aseptic glenoid loosening and superior cuff failure had a higher CSA than these without required revision surgery. This finding is further supported by the 2023 study conducted by Adebol et al. [19], which analyzed factors influencing the development of secondary rotator cuff insufficiency following anatomic shoulder arthroplasty. Their study identified increased glenoid inclination, a higher CSA, an oversized humeral component, a thicker glenoid component, and more advanced fatty infiltration of the rotator cuff muscles as significant risk factors. The causes of revision were recorded in this study; however, they were not included in the subgroup analysis of failure rates due to small sample sizes. However, among the revision cases, the majority of patients (*n* = 6; 55%) experienced isolated glenoid loosening, while two patients presented with a combination of glenoid loosening and secondary rotator cuff insufficiency as the reasons for revision. When reviewing the literature on AHD, our findings show only partial agreement. It is well established that the development of cuff tear arthropathy in the native joint, due to rotator cuff insufficiency, leads to a subsequent cranial migration of the humeral head and a reduction in AHD [40]. Similar patterns are expected with secondary rotator cuff insufficiency in patients with TSA, as demonstrated in previous studies [41–43]. The lack of influence of AHD on revision rates in our cohort might be explained by the fact that most revisions were due to glenoid loosening rather than secondary rotator cuff insufficiency. Another interesting finding is that osteolysis was observed to a similar extent (Group A: 47%, Group B: 45%) in both groups. However, only 45% of surgical revised cases presented with osteolysis. This supports the existing evidence in the literature that radiographically detected osteolysis in general does not necessarily lead to revision after TSA only when they occur across multiple zones [44]. This correlation is further amplified if osteolysis are located in distal zones and the number of involved zones rises [44].

This study is not without limitations. In addition to its retrospective design, the relatively small cohort size must be acknowledged. However, this was due to strict inclusion and exclusion criteria, which enhance the quality and relevance of our findings. The study aimed to analyze implant longevity and revision rates after TSA, with a focus on potential risk factors. Although the specific causes of revision were documented, they were not analyzed due to the limited sample size, which restricts our ability to draw conclusions regarding the influence of individual risk factors on specific revision causes in TSA. Moreover, the exclusion of early revisions does not allow for any statement on complications within the first year after surgery. The benefit is the focus on implant performance rather than perioperative complications. Additionally, follow-up imaging relied solely on conventional radiographs, as we adhered to standardized postoperative protocols without performing CT scans.

## Conclusions

The findings of this study weaken the potential impact of preoperative anatomical factors. Only the humeral head-stem index (HSI) had a minor implant longevity and revision risk in total shoulder arthroplasty (TSA). An increased HSI was associated with higher revision rates, even when anatomical restoration of the centre of rotation (COR) was achieved. However, the small absolute difference in HSI between groups raises questions about the clinical significance of this finding. These insights underscore the critical role of careful preoperative planning and component selection in improving TSA outcomes. This study found no significant impact of radiographic properties on implant survival except the HSI. The impact of the HSI however, encourage further investigation into optimal sizing to reduce revision rates.

## Data Availability

The data that support the findings of this study are not openly available due to reasons of sensitivity and are available from the corresponding author upon reasonable request. Data are located in controlled access data storage at Department of Orthopaedic Surgery, Julius-Maximilians University Wuerzburg, Koenig-Ludwig-Haus, Brettreichstrasse 11, 97074 Wuerzburg, Germany.
